# Investigating EFL students’ engagement in project-based speaking activities: from a multi-dimensional perspective

**DOI:** 10.3389/fpsyg.2025.1598513

**Published:** 2025-06-25

**Authors:** Jianer Zhong, Lilliati Ismail, Yueqiao Lin

**Affiliations:** ^1^Department of Foreign Languages, Yangjiang Polytechnic, Yangjiang, Guangdong, China; ^2^Department of Language and Humanities Education, Universiti Putra Malaysia, Serdang, Selangor, Malaysia

**Keywords:** behavioral engagement, emotional engagement, cognitive engagement, agentic engagement, project-based learning, speaking activities

## Abstract

**Introduction:**

Student engagement is essential for improving academic performance and achievement. Project-Based Learning (PBL) has emerged as a promising instructional approach to enhance student engagement. However, its effectiveness across various engagement dimensions remains under-explored.

**Methods:**

This study employed a quasi-experimental mixed-methods approach to examine the impact of PBL on student engagement, with a specific focus on its behavioral, emotional, cognitive, and agentic dimensions. It involved 96 first-year students from a Chinese polytechnic, who were assigned to either an experimental group (*n* = 49) or a control group (*n* = 47). While the control group received instruction through conventional teaching methods, the experimental group engaged in PBL. Data were collected through a combination of questionnaires and interviews.

**Results:**

Findings indicate that PBL significantly enhances emotional, behavioral, and cognitive engagement. However, the results also reveal that PBL had no significant effect on agentic engagement. Students perceived PBL as a highly effective method for enhancing engagement, as evidenced by increased strong interest and enjoyment, focused concentration and effort, active use of cognitive strategies, and proactive contribution to learning.

**Discussion:**

This study demonstrates the potential of PBL to foster deeper engagement in EFL settings, particularly in behavioral, emotional, and cognitive dimensions. Nonetheless, its limited effect on agentic engagement suggests a need for instructional adjustments that encourage student autonomy and voice. These findings provide practical insights for educators aiming to implement PBL effectively in EFL classrooms.

## 1 Introduction

Student engagement is crucial for promoting proactive learning and academic performance (Lei et al., 2018). It is a multiple-dimensional concept that includes different interconnected components of behavioral, cognitive, emotional, and agentic engagement (Reeve and Tseng, 2011). Throughout learning activities, students manifest varying degrees of engagement, which can serve as a predictive indicator of their academic success (Wong and Liem, 2022). Students who actively participate in learning activities show a positive link with higher academic achievement compared to those who do not participate actively (Alqurashi, 2022). Augmenting engagement in the educational process is believed to yield heightened learning efficacy. To foster deep and sustained engagement, educators must design learner-centered, interactive activities that promote autonomy, collaboration, and meaningful communication.

Despite these pedagogical imperatives, many college students pursuing English as a foreign language (EFL) continue to experience disengagement, particularly in English-speaking classes. English instruction is often dominated by conventional teaching methods (CTM), which are teacher-centered and emphasize rote learning and mechanical speaking drills. These methods offer limited opportunities for meaningful interaction or self-expression. As a result, learners frequently engage in passive participation and exhibit reluctance to speak in English (Azeez, 2023). Such passivity undermines their confidence and leads to poor oral proficiency outcomes. In contrast, the growing emphasis on communicative competence and real-world application in EFL pedagogy underscores the need for more dynamic and student-driven instructional approaches.

Project-based learning has emerged as a promising alternative to CTM, particularly for enhancing student engagement (Novianti and Kusumayanthi, 2023; Imbaquingo and Cárdenas, 2023). PBL is an instructional method where students gain knowledge and skills through active engagement in real-world projects (Buck Institute for Education, 2022). Unlike CTM, PBL serves as a promising pedagogical approach, as its learner-centered and inquiry-driven nature inherently supports various dimensions of engagement (Torres and Rodríguez, 2017). PBL engages students in meaningful, real-world tasks that involve collaboration, reflection, and active use of the target language. This approach supports language development while also fostering autonomy, motivation, and critical thinking, which are the essential factors for long-term academic success in EFL learning (Makkonen et al., 2021; Zhang and Ma, 2023).

Although existing research suggests that PBL can improve student engagement (Morais et al., 2021; Turcotte et al., 2022), its impact within EFL and English as a Second Language (ESL) contexts remains under-explored and inconclusive (Kusumawati, 2019; Hairuddin and Irmawati, 2024). Most studies have treated student engagement as a singular construct in the framework of PBL, neglecting its multidimensional nature. This gap is particularly pronounced in EFL contexts, where classroom engagement is often narrowly conceptualized and measured. Yet, understanding and fostering multidimensional engagement is crucial in EFL pedagogy, as it provides a more holistic view of how learners interact with content, peers, and the learning process in the PBL environments. Consequently, there is a pressing need to investigate how PBL influences distinct facets of engagement (behavioral, cognitive, emotional, and agentic), particularly in the context of Chinese EFL classrooms where passive learning traditions remain prevalent.

This study aimed to investigate whether PBL could significantly enhance EFL students’ engagement across all four dimensions in English-speaking classes when compared to CTM. It also explored how the EFL students perceived the implementation of PBL. The study was guided by the two primary research questions:

1. Does PBL have more effects than CTM on EFL students’ behavioral, emotional, cognitive, and agentic engagement?
2. What are EFL students’ perspectives on the implementation of PBL in enhancing the four engagement dimensions?

## 2 Literature review

### 2.1 Framework of student engagement

The concept of student engagement has been extensively examined in educational research for over 70 years (Kuh, 2009) and is widely recognized as a multifaceted construct reflecting students’ active involvement in learning (Fredricks et al., 2019). Early models emphasized behavioral and emotional components (Finn, 1989), with cognitive engagement later introduced to reflect students’ investment in learning strategies and deep processing (Connell and Wellborn, 1991). This three-dimensional framework became the dominant paradigm (Fredricks et al., 2004; Wang et al., 2011). However, Reeve and Tseng (2011) proposed the addition of agentic engagement, highlighting students’ proactive contributions to shaping their learning environment, such as asking questions, expressing preferences, and suggesting improvements, thereby extending the framework to four dimensions.

This study adopts Reeve and Tseng’s (2011) four-dimensional model, encompassing behavioral, emotional, cognitive, and agentic engagement. Behavioral engagement refers to students’ attention, effort, and persistence in academic tasks (León et al., 2015), while emotional engagement captures their affective responses to learning, including feelings of interest, enjoyment, and belonging (Bond and Bedenlier, 2019). Cognitive engagement involves the use of sophisticated learning strategies and a commitment to mastering content (Reeve, 2013), and agentic engagement reflects students’ intentional efforts to influence instruction and optimize their learning experience (Reeve, 2012; Bandura, 2018).

**Figure 1:**
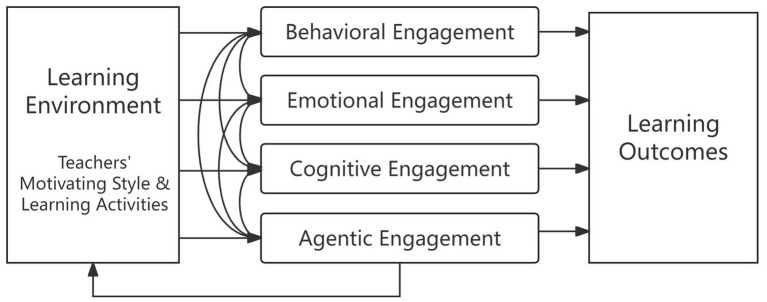
Four interrelated dimensions of student engagement (Reeve and Tseng, 2011).

The four dimensions of student engagement are dynamically interconnected, contributing to positive learning outcomes (see Figure 1). A supportive learning environment, enriched by a teacher’s motivational approach and engaging activities, promotes students’ behavioral, emotional, cognitive, and agentic engagement, resulting in positive learning outcomes (Reeve and Tseng, 2011). Emotional and cognitive engagement can influence behavioral participation, while agentic engagement often fosters a more supportive and interactive learning environment. Classrooms that provide motivational support and stimulating tasks are more likely to enhance all four engagement dimensions, promoting deeper learning and sustained academic growth (Reeve, 2013).

### 2.2 Student engagement in PBL within EFL and ESL contexts

Research on student engagement in PBL has garnered increasing attention in within EFL and ESL contexts (Hairuddin and Irmawati, 2024; Li et al., 2024). However, compared to other educational domains, the volume and depth of research in this area remain limited. Much of the existing literature consists of exploratory studies focusing on students’ perceptions of PBL rather than offering rigorous evaluations of its impact on engagement. For instance, Putri et al. (2017) employed observation, interviews, and open-ended questionnaires to explore student engagement in PBL at an Indonesia school, finding generally positive responses. Similarly, Park and Eisenhower (2019) used observational methods and interviews to evaluate Korean EFL learners’ experiences with a language program, concluding that students perceived PBL as beneficial for language learning. While such studies provide valuable insights into learners’ subjective experiences, their reliance on self-reported data and the absence of control groups limit the validity and generalizability of their findings.

Other research has attempted to measure the influence of PBL on student engagement using a variety of tools, including questionnaires and observation sheets (Aubrey, 2022; Imbaquingo and Cárdenas, 2023; Hairuddin and Irmawati, 2024). For example, Novianti and Kusumayanthi (2023) implemented a collaborative PBL program in an EFL speaking class and reported increased student participation. Al-Bahadli et al. (2023) found similarly positive outcomes using questionnaires to assess engagement among Ukrainian college students. However, these studies often lacked longitudinal data and comparative designs, raising concerns about internal validity and the sustainability of engagement gains. Many also failed to control for variables such as language proficiency, motivation, or prior learning experiences, which may have influenced the outcomes.

Importantly, not all studies report uniformly positive effects. Johnson and Delawsky (2013), in a quasi-experimental design involving checklists, attendance logs, surveys, and test scores, found that PBL had no significant impact on emotional engagement and yielded inconclusive results on cognitive engagement. Moreover, it appeared to reduce behavioral engagement. Zheng et al. (2022) conducted a case study of five undergraduate students engaged in collaborative writing projects and observed declining engagement over time, with some students transitioning from active participants to passive “free riders.” These findings highlight the potential for disengagement in group-based PBL activities, especially when group dynamics or task structures are poorly managed.

Taken together, these mixed results suggest that the impact of PBL on student engagement in EFL and ESL contexts is far from settled. The diversity in methodological approaches, ranging from qualitative case studies to cross-sectional surveys, has produced inconsistent findings, limiting the ability to draw generalizable conclusions. Moreover, a critical gap in the literature is the lack of studies that investigate student engagement from a multi-dimensional perspective. Although engagement is widely recognized as encompassing behavioral, cognitive, emotional, and agentic dimensions (Reeve and Tseng, 2011), only a few studies have explored more than two of these simultaneously. Zheng et al. (2022) tracked changes in behavioral, emotional, and cognitive engagement during collaborative writing tasks, while Li et al. (2024) focused on emotional and social engagement over time. Zhong et al. (2024) examined all four dimensions in the context of EFL speaking classes but relied solely on self-report data without a comparison group, limiting the strength of causal claims. These studies, while important, often suffer from methodological constraints such as small sample sizes, lack of experimental controls, and an over-reliance on subjective instruments.

In light of these limitations, further empirical research is needed to assess the effects of PBL on multidimensional engagement in EFL speaking classes using robust comparative designs. To address this gap, the present study investigates the impact of PBL on behavioral, cognitive, emotional, and agentic engagement, compared to CTM. It also seeks to explore learners’ perceptions of PBL to provide a more nuanced understanding of how this approach may foster or hinder active engagement in language learning.

## 3 Methodology

### 3.1 Research design

This study utilized mixed methods with a quasi-experimental design incorporating both quantitative assessments and qualitative interviews. The quasi-experimental design was used to examine changes in the dependent variable after the intervention without relying on random assignment (Finn and Krysik, 2013). Quantitative methods were employed to evaluate the impact of PBL on Chinese EFL students’ engagement, while qualitative methods explored their perspectives on implementing PBL. Together, these methods provided a holistic understanding of the effectiveness of PBL in enhancing student engagement.

### 3.2 Participants

The study involved a homogeneous group of 96 first-year EFL students from a Chinese polytechnic. The group included 92 females and 4 males between the ages of 17 and 21 years. All students involved were native Mandarin Chinese speakers who specialized in English education. *A priori* power analysis was conducted using G*Power 3.1 to determine the appropriate sample size for detecting a medium effect size (*f* = 0.25) with a power of 0.80 and an alpha level of 0.05, which indicated a minimum total sample size of 86 participants. The final sample of 96 exceeded this requirement, ensuring adequate statistical power for the planned analyses.

Participants were separated into an experimental group (*N* = 49) and a control group (*N* = 47), with efforts made to ensure comparable baseline characteristics, including prior academic performance and demographic factors. The experimental group was taught using PBL, while the control group received instruction through CTM. To gain deeper insights, 10 participants from the experimental group, selected based on their ability to provide detailed interview responses, were interviewed. The participants were given pseudonyms to ensure confidentiality.

### 3.3 Instruments

Questionnaires were completed to evaluate the effects of PBL on the four dimensions of engagement. The Student Classroom Engagement (SCE) scale, developed by Reeve in 2013, was utilized to assess emotional, behavioral, cognitive, and agentic involvement. The SCE scale consists of 21 questions in total, including 5 questions dedicated to emotional engagement, 5 questions dedicated to behavioral engagement, 4 questions dedicated to cognitive engagement, and 7 questions dedicated to agentic engagement. Participants needed 20–30 min to complete the questionnaire. The participants provided subjective assessments of their level of classroom participation using a Likert scale, which extended from 1 (strong disagreement) to 7 (strong agreement). Considering that the Chinese version of the SCE scale is easier for participants to understand, a back-to-back translation was conducted. The researcher translated the questionnaire into Chinese. More English back-translations were conducted by two English teachers who were native Chinese but proficient in English as well. The teachers engaged in discussions to resolve any disagreements that arose, ultimately reaching consensus on the translation.

Semi-structured interviews were conducted after the implementation of PBL. The interviews were conducted to explore students’ perceptions of using PBL in improving student engagement. The interview questions were created by the researchers in accordance with the research objectives and questions. The interview was structured around six primary questions (see Appendix Table A1). The questions were subjected to evaluation and validation by two educational experts in the relevant field prior to conducting the interviews. In the interview, students were guided by, but not limited to, the list of questions. The interview was carried out under informal circumstances to encourage students to share genuine ideas on PBL in Chinese without worrying about leaving an unfavorable impression on the interviewer or influencing the final scores of the course.

### 3.4 Intervention

**Figure 2:**
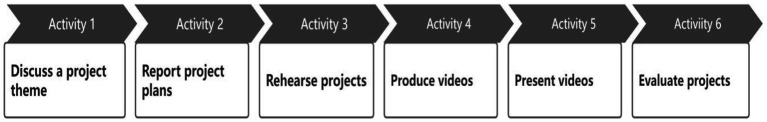
PBL activities.

Based on the essential elements of project design (Larmer et al., 2015), the study incorporated the following six specific PBL activities: (a) Discuss a project theme. The teacher introduced the project theme by posing a question. Students engaged in group discussions to determine their respective project themes. (b) Report project plans. Students orally presented their project plans in the classroom. (c) Develop projects. Students gathered information from various sources, including libraries, the internet, interviews, questionnaires, and field trips. (d) Produce videos. Students created films, wrote screenplays, recorded conversations for the video products, and ultimately produced videos. (e) Present videos. Each group presented their videos in the classroom and addressed questions from both the instructor and their peers. (f) Evaluate projects. The teacher, in collaboration with the students, evaluated their project work and provided feedback (see Figure 2).

**Figure 3:**
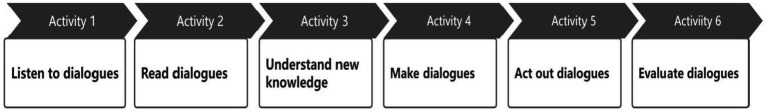
CTM activities.

Compared to PBL, CTM involved six activities. (a) Listen to dialogs. At the start of class, the teacher played exemplar dialogs from an audio recording. The students attentively listened to the dialogs, with a specific emphasis on the correct pronunciation and intonation. (b) Read dialogs. Students read the dialogs, either in conjunction with the recording or in collaboration with their partners. (c) Understand new knowledge. The teacher provided guidance on how to incorporate the newly learned vocabulary and phrases into the talks. Students attentively absorbed and endeavored to comprehend novel knowledge and skills. (d) Develop dialogs. Students were instructed to make dialogs and participate in role-plays with their partners, applying the newly acquired knowledge and skills to specific circumstances. (e) Act out dialogs. Several students were mandated to adopt various characters and perform the dialogs in front of the class. (f) Evaluate dialogs. The teacher and students assessed the presentations (see Figure 3).

### 3.5 Procedures

The experiment spanned one semester, specifically 14 weeks, commencing in late September and concluding in late December (see Table 1). The participants received instruction from two Chinese instructors, each boasting over a decade of experience teaching English. To minimize instructor-related bias, both instructors were randomly assigned to teach either the experimental or control group, and they were not involved in the assignment process. The same set of instructional materials and assessment criteria were used across both groups to ensure consistency. The experimental group was taught using PBL, while the control group received instruction through CTM. Prior to the intervention, both instructors participated in a calibration session to align on instructional delivery and assessment standards. Furthermore, inter-rater reliability was established through double-scoring a random subset of students’ samples of academic performance, resulting in a Cohen’s kappa of 0.81, indicating strong consistency between the raters.

**Table 1:** Procedures of the study.

Time	PBL	CTM
Week 1	Questionnaire	Questionnaire
Week 2–4	Project 1: What are the qualities of an excellent college student?	Topic 1: Describing a friend
Week 5–7	Project 2: What are the attractions of a location?	Topic 2: Describing a place
Week 8–10	Project 3: Which are ideal jobs for college students?	Topic 3: Describing a job
Week 11–13	Project 4: How do you receive visitors?	Topic 4: Describing an experience
Week 14	Questionnaire Interview	Questionnaire

During the first week, both sets of participants were instructed to fill out the questionnaire. From the second week through the twelfth week, distinct treatments were administered to the different groups. The experimental group engaged in PBL. The participants were organized into eight groups, with each group comprising six to seven members. The groups then engaged in the exploration of four inquiry-based projects. (a) What are the qualities of an excellent college student? (b) What are the attractions of a location? (c) Which are ideal jobs for college students? (d) How do you receive visitors? Students needed to complete each project by engaging in six project-based speaking activities within 3 weeks. In the control group, CTM was employed. In contrast to the projects used in PBL, the topics covered in the CTM class were: (a) Describing a friend; (b) Describing a place; (c) Describing a job; and (d) Describing an experience. Each topic was taught for 3 weeks. The control group followed CTM instruction.

At the end of the last week, all students were asked to complete the same questionnaire once again. Additionally, in the experimental group, 10 students were selected for interviews to further understand the perceptions of using PBL to promote student engagement.

### 3.6 Data analysis

The questionnaire data from both groups were analyzed using SPSS 27.0. A reliability analysis was conducted to assess the internal consistency. The results indicated that the Cronbach’s Alpha coefficients for emotional, behavioral, cognitive, and agentic engagement were 0.926, 0.914, 0.941, and 0.953. The overall scale exhibits a Cronbach’ s Alpha of 0.962, and all results fall within the range of 0 to 1, denoting a high degree of reliability. Subsequently, a normality assessment was performed on the scale by examining Skewness values. The findings demonstrate that the Skewness values of the questionnaires fall within the range of −2 to +2, signifying a normal distribution of the test scores. Ultimately, a one-way MANOVA test was executed to compare the average scores of the four engagement dimensions between the two groups. The one-way MANOVA test was employed to measure the effects of the independent variable on several dependent variables (George and Mallery, 2024). This helped to determine whether there were significant differences in behavioral, emotional, cognitive, and agentic engagement between the PBL and CTM groups.

The semi-structured interviews were recorded and then transcribed in their entirety. The transcripts were given back to the participants for member verification, a process designed to ensure the accuracy of the transcripts in capturing the participants’ intended messages. As the transcripts were in Chinese, a double-back translation of the interview questions and transcripts was also performed to enhance validity. The researcher translated the transcripts into English, which were then reviewed and improved by the other two professional translators. Afterward, the interview transcripts were analyzed using NVivo 12.0 and were subjected to thematic analysis (Braun and Clarke, 2006). The procedures involved six steps: familiarizing oneself with the data, creating initial codes, identifying categories or themes, examining all the codes, categories, and themes, evaluating and refining the themes, and finally generating the report. Two proficient raters conducted the coding of the interview data. The level of agreement between the raters was found to be 83%. Conflicts that emerged during the coding process were addressed through collaborative discussions, ultimately reaching a consensus.

## 4 Results

### 4.1 The effects of PBL on student engagement

Descriptive statistics were utilized to examine the pretest and posttest questionnaire scores. Table 2 shows the mean and standard deviation across the four dimensions for both groups. In the PBL group, the mean of behavioral (*M* = 4.617, SD = 0.523), emotional (*M* = 4.106, SD = 0.586), cognitive (*M* = 3.969, SD = 0.618), and agentic (*M* = 3.637, SD = 0.616) engagement at the posttest were higher than those of behavioral (*M* = 3.820, SD = 0.614), emotional (*M* = 3.714, SD = 0.698), cognitive (*M* = 3.714, SD = 0.736), and agentic (*M* = 3.612, SD = 0.298) engagement at the pretest. In the CTM group, the posttest mean for behavioral (*M* = 3.830, SD = 0.384), emotional (*M* = 3.804, SD = 0.513), cognitive (*M* = 3.713, SD = 0.549), and agentic (*M* = 3.623, SD = 0.401) engagement surpassed the pretest mean for behavioral (*M* = 3.787, SD = 0.535), emotional (*M* = 3.745, SD = 0.721), cognitive (*M* = 3.707, SD = 0.706), and agentic (*M* = 3.609, SD = 0.276) engagement. The results show that students’ engagement in both groups increased in the posttest.

**Table 2:** Cumulative mean and standard deviation of the four engagement dimensions.

Engagement	Group	Pretest		Posttest	
		Mean	SD	Mean	SD
Behavioral	PBL	3.820	0.614	4.617	0.523
	CTM	3.787	0.535	3.830	0.384
Emotional	PBL	3.751	0.698	4.106	0.586
	CTM	3.745	0.721	3.958	0.569
Cognitive	PBL	3.714	0.736	3.969	0.618
	CTM	3.707	0.706	3.713	0.549
Agentic	PBL	3.612	0.298	3.637	0.616
	CTM	3.609	0.276	3.613	0.401

**Figure 4:**
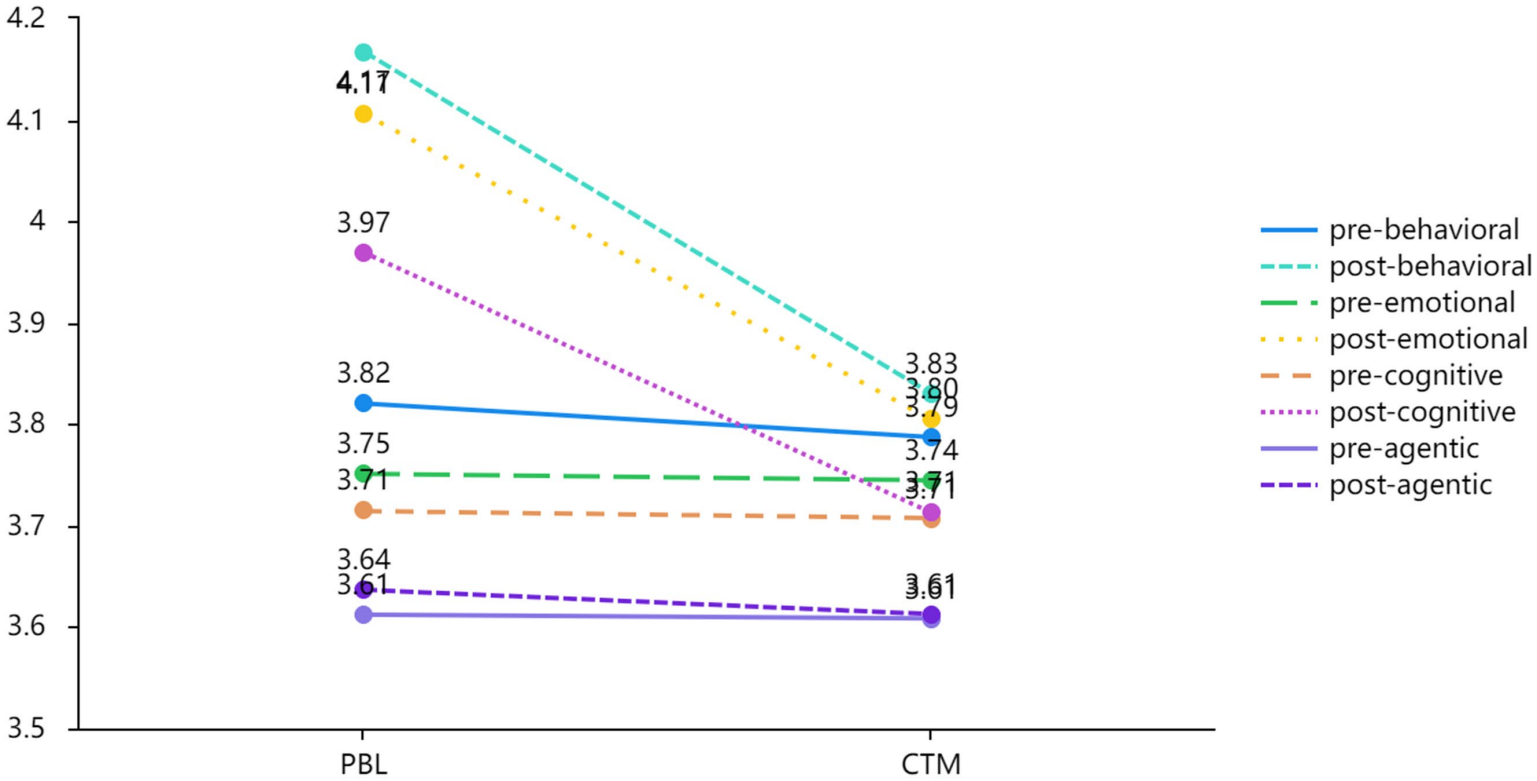
Comparison of the four dimensions within the PBL and CTM.

Figure 4 illustrates the changes in mean scores for the four dimensions from the pretest to the posttest among the PBL and CTM groups. The results reveal a significant increase in the mean scores for both behavioral and emotional engagement between the pretest and posttest in both the PBL and CTM groups. In contrast, only minor improvements were noted in cognitive and agentic engagement between the two tests for the two groups. These findings suggest that significant enhancements varied in the four dimensions of engagement among the PBL and CTM groups.

A one-way MANOVA test was performed to examine the impact of PBL on student engagement in comparison to CTM before and after using PBL. The significance values of the pretest scores for behavioral (*p* = 0.779), emotional (*p* = 0.965), cognitive (*p* = 0.963), and agentic engagement (*p* = 0.949) all exceed 0.05, indicating that there were no significant differences between the two groups across these four dimensions prior to implementing PBL. Box’s test was conducted on the posttest mean scores to assess the homogeneity of covariance matrices within the one-way MANOVA procedures. The resulting significance value of 0.175 indicated that the assumption of homogeneity was satisfied. Additionally, Levene’s test was performed to evaluate the equality of error variances. The results confirmed the assumption of equal error variances, with significance values for behavioral (*p* = 0.055), emotional (*p* = 0.175), cognitive (*p* = 0.804), and agentic (*p* = 0.604) engagement all meeting the criteria.

Table 3 presents the results of comparing student engagement between the PBL and CTM groups in the posttest. A significant difference was found between the PBL and CTM groups in behavioral engagement [*F*(1, 94) = 12.882, *p* = 0.001]. The partial was *η*^2^ 0.121, indicating a medium to large effect size. The PBL group (*M* = 4.167, SD = 0.523) reported significantly higher behavioral engagement than the CTM group (*M* = 3.803, SD = 0.384). The findings indicate that PBL meaningfully enhanced students’ behavioral participation in speaking activities. Emotional engagement also showed a significant difference between the two groups [*F*(1, 94) = 7.183, *p* = 0.009]. The partial *η*^2^ was 0.071, reflecting a medium effect size. The PBL group (*M* = 4.106, SD = 0.586) outperformed the CTM group (*M* = 3.804, SD = 0.513). These findings indicate that students in the PBL condition experienced greater enjoyment and interest in learning. Regarding cognitive engagement, there was a statistically significant difference between groups [*F*(1, 94) = 4.612, *p* = 0.034]. The partial *η*^2^ was 0.047, indicating a small to medium effect size. The PBL group (*M* = 3.969, SD = 0.618) scored higher than the CTM group (*M* = 3.713, SD = 0.549), with a 95% CI for the mean difference ranging from 0.018 to 0.489. These findings indicate that PBL supported deeper cognitive investment in learning tasks.

**Table 3:** Comparison of student engagement between groups in the posttest.

Engagement	Group	*N*	Mean	SD	*F*	Sig.	Partial eta squared
Behavioral	PBL	49	4.167	0.523	12.882	0.001**	0.121
	CTM	47	3.830	0.384			
Emotional	PBL	49	4.106	0.586	7.183	0.009**	0.071
	CTM	47	3.804	0.513			
Cognitive	PBL	49	3.969	0.618	4.612	0.034*	0.047
	CTM	47	3.713	0.549			
Agentic	PBL	49	3.637	0.401	0.051	0.823	0.001
	CTM	47	3.613	0.618			
Overall					5.111^b^	0.001**	0.183

**p < 0.01, *p < 0.05.

However, agentic engagement did not show a statistically significant difference between the two groups [*F*(1, 94) = 0.051, *p* = 0.823, partial *η*^2^ = 0.001]. Although the PBL group (*M* = 3.637, SD = 0.401) had a slightly higher mean than the CTM group (*M* = 3.613, SD = 0.618). This result contrasts with qualitative findings, where several students in the PBL group reported proactive behaviors. This discrepancy may be due to the limitations of the survey instrument in capturing context-specific expressions of agency or to the possibility that agentic behaviors require a longer intervention period to become observable at scale.

Overall, a multivariate test revealed a significant difference in overall student engagement between the two groups, *F*(4, 91) = 5.111, *p* = 0.001, with a large effect size (partial *η*^2^ = 0.183). These results indicate that PBL is effective in promoting student engagement across multiple dimensions, particularly behavioral, emotional, and cognitive aspects.

### 4.2 Perspectives on the implementation PBL to enhance student engagement

The semi-structured interviews were further employed to explore how EFL students perceived the implementation of PBL to enhance student engagement. Four themes emerged from the data, including strong interest and enjoyment, focused concentration and effort, active application of cognitive strategies, and proactive contribution to learning. To facilitate a systematic presentation, these four themes are distinctly explained.

#### 4.2.1 Strong interest and enjoyment

The initial focus on enhancing student engagement revolved around generating interest and enjoyment, specifically emphasizing emotional engagement. This refers to the presence of emotions that encourage active involvement in projects. Out of the 10 participants, six (6/10) expressed their interest in the project themes, which served as a motivating factor for their active participation in the learning process. Chen and Liu reported:

*“I was most interested in the project theme of introducing scenic spots. Because it allowed us to understand a place better, for example, when we went to film on Hailing Island, it not only enriched our lives by providing an activity outside of our regular classes, but also allowed us to relax and enjoy ourselves.”* (Chen, P3).

*“I was keen on the project of receiving visitors. It involved conversing with guests using appropriate methods. From this project, I learned many different meanings of expressions, such as the word ‘anytime’ that the teacher taught in class. It could be used in conversations with guests to indicate a willingness to serve at any time, which I found quite interesting.”* (Liu, P2).

The participants highlighted “introducing scenic spots” and “receiving visitors” as captivating project themes. They showed a particular preference for the theme of introducing scenic spots, as it provided an opportunity to explore the location while completing the project. Similarly, they found the theme of receiving visitors engaging because it enabled them to learn practical expressions.

Meanwhile, five participants (5/10) expressed keen interest and enjoyment during the project discussion and execution phases. They derived significant pleasure and satisfaction from engaging in project activities. Gong and Li mentioned:

*“I was quite interested in discussing topics because it involved all the group members gathering together, sharing their opinions, and having lively discussions with different perspectives. It was interesting to me because everyone could express their own opinions and viewpoints, and execute projects.”* (Gong, P8).

*“During the discussion phase, I enjoyed hearing everyone’s ideas and finding common ground. Planning the project, allocating tasks, and executing it were also fascinating. When we executed the project, we realized discrepancies between our plans and reality. For instance, when we invited someone to dinner over the phone, as outlined in our script, we found that we needed to improve our English communication skills, requiring further discussion and refinement. I found this process very engaging.”* (Li, P5).

The participants valued the discussion phase of the topics, as it gave them a chance to share their views and exchange a variety of ideas. They also enjoyed the project execution phase, seeing it as a valuable opportunity to enhance their communication skills. The results suggest that the PBL method has the potential to enhance students’ emotional engagement by stimulating their interest and enjoyment through captivating themes and project activities.

#### 4.2.2 Focused concentration and effort

The second theme highlighting the improvement of student engagement is characterized by focused concentration and effort. This theme is linked to behavioral engagement, which entails students’ attentive focus and sustained dedication. Participants (6/10) emphasized their active participation and concentrated focus during classroom project activities. Gong and Chen stated:

*“When the teacher explained what the project was about, I actively participated in our group meetings. For example, when we discussed topics related to our theme, I would express my opinions and viewpoints actively. When I was assigned tasks, I would complete them diligently and conscientiously.”* (Gong, P8).

*“After the presentations, during the question-and-answer session, my participation was also high. If I saw other groups presenting videos and did not understand something, we would ask them and listen to their answers or suggestions. If my group leader was questioned by other groups, whether I was presenting or not, I would be very active, constantly thinking about how to answer their questions, so my participation was very high.”* (Chen, P3).

The participants exhibited strong involvement in topic discussions, task completion, and question-and-answer sessions during class. Their engagement extended beyond the classroom as they continued discussions with group members afterward, reflecting a consistent commitment to learning.

The participants (4/10) also described their continuous dedication, both in and out of the classroom, to ensure the successful completion of projects. Gan said:

“*I would listen carefully to the teacher in class. After class, I would take out my oral English book, review the project, and if I encountered any problems or had any suggestions, I would discuss them with my group members to further improve our project work.” (Gan, P6).*

Participants (5/10) believed that group dynamics motivated them to focus and dedicate effort to project activities. Feng reported:

“*If we wanted to be lazy, we did not have time for it because everyone else was working, and it was not good to be the only one slacking off. For example, when discussing a topic, everyone expressed their opinions. If we were just sitting there silently, it felt strange, like you were being lazy and not genuinely participating, which did not feel right, so you ended up speaking up.”* (Feng, P1).

The participants cultivated a strong sense of belonging while working in groups, feeling compelled to contribute actively instead of staying passive. This sense of responsibility encouraged their active participation and discouraged complacency. These findings suggest that behavioral engagement improved as they demonstrated greater concentration and exerted more effort in project-based learning.

#### 4.2.3 Active application of cognitive strategies

The third theme identified in enhancing student engagement was the active application of cognitive learning strategies. It is related to cognitive engagement, which involves the utilization of sophisticated, deep, and personalized learning approaches. The participants often utilized association and summarization strategies to support their cognitive learning during the PBL process. Participants (6/10) endeavored to employ an association strategy to internalize the new knowledge acquired through the PBL method. Chen reported:

“*We had some prior knowledge about what to pay attention to during job interviews, but it wasn’t comprehensive enough, so we sought out new knowledge to understand more about what to pay attention to during job interviews.”* (Chen, P3).

Apart from the association strategy, participants (4/10) used summarization strategies to draw conclusions on what they knew and learned. Feng mentioned:

*“I reviewed the content learned on the same day and summarized it in my mind or on paper.”* (Feng, P1).

As illustrated above, the participants employed association and summarization strategies to deepen their understanding of the learning materials. These approaches not only improved retention but also helped integrate new concepts with prior knowledge, fostering a more thorough comprehension of the subject matter. These findings reveal that students’ cognitive engagement improved as they employed certain cognitive learning strategies to acquire new knowledge during the PBL process.

#### 4.2.4 Proactive contribution to learning

The fourth theme concerning the enhancement of student engagement revolves around proactive contributions to the flow of learning. This theme is associated with agentic engagement, which entails actively participating in learning activities and enhancing the learning process rather than passively receiving knowledge. Participants (5/10) noted that they actively facilitated learning in class through interactions with the teacher, such as asking and answering questions. Participants mentioned that they proactively sought clarification from the teacher by asking questions or requesting assistance when they encountered difficulties. Chen (P3) stated:

*“I actively raised my hand to ask questions in class. After each project presentation, there was a question session during which I asked questions.”* (Chen, P3).

Participants (4/10) expressed that they willingly volunteered to actively respond to teachers. Feng claimed:

*“I was always the first to raise my hand to answer questions. Actually, I was quite nervous, but I wasn’t afraid. I could bravely express myself.”* (Feng, P1).

The findings suggest most participants actively contributed to a positive learning environment in the classroom. They sought clarification when facing difficulties, showing initiative in their learning. Additionally, they expressed a willingness to volunteer responses, with some overcoming nervousness to confidently participate in class discussions.

However, not all participants exhibited high levels of agentic engagement, revealing a more nuanced picture. Two participants, in particular, described a more passive stance during classroom interactions. Liang and Jiang mentioned:

*“I listened to what the teacher says, but I might not be very proactive in learning.”* (Liang, P4).

*“I actively thought about the teacher’s questions, but I did not always speak up.”* (Jiang, P10).

These responses highlighted a form of agentic engagement that did not necessarily translate into outward participation. Their reluctance to vocalize their thoughts or take initiative in class may stem from deeply embedded cultural norms. In a collectivist educational setting like China’s, where maintaining group harmony and deference to authority are often valued, students may avoid assertive behaviors that could be perceived as disruptive or disrespectful. Additionally, the traditional teacher-centered hierarchy might discourage learners from challenging or interrupting the teacher, even within a PBL framework. These contextual factors suggest that agentic engagement in such settings may be expressed in more subtle or indirect ways, and thus may not be fully captured through standard engagement measures.

## 5 Discussion

This study sought to investigate the effects of PBL on student engagement among college EFL students from a multi-dimensional perspective. The findings of this study revealed that a significant difference in overall student engagement was observed between the PBL and CTM groups. The differences between the two methods were prominently found in EFL students’ behavioral, emotional, and cognitive engagement. However, no significant differences were found in agentic engagement between the PBL and CTM groups. The findings also showed that PBL was viewed as an effective instructional method for boosting student engagement, supported by the four themes of strong interest and enjoyment, focused concentration and effort, active application of cognitive strategies, and proactive contribution to learning. These findings indicated that EFL students experienced tangible enhancements through the implementation of PBL. These findings align with the results of certain other studies (Mercer and Dörnyei, 2020; Novianti and Kusumayanthi, 2023; Li et al., 2024), which highlighted the potential of PBL in facilitating learning processes and improving student engagement when compared to CTM. This study builds on prior research by investigating the effects of PBL in promoting emotional, behavioral, cognitive, and agentic engagement, which is a relatively under-explored area in EFL and ESL literature.

Regarding emotional engagement, the findings reveal that students participating in PBL demonstrated greater emotional engagement in their learning compared to the CTM group. Emotional engagement is associated with the presence of positive emotions, such as interest, enjoyment, and satisfaction (Reeve, 2012). One possible explanation for the higher emotional engagement in PBL could be the inclusion of intriguing project topics. PBL offers students the opportunity to work on authentic and relevant projects, which can capture their interests more effectively than the CTM approach (Gabuardi, 2021). The project topics explored in this study are closely tied to students’ academic and personal experiences, igniting their enthusiasm for learning. As freshmen, students were particularly intrigued by these topics, as they sought to familiarize themselves with college life and share their aspirations with peers. Through interviews, participants expressed genuine enthusiasm for the project themes. Consequently, these interesting topics serve as catalysts for emotionally engaging students in PBL sessions (Liu and Zuo, 2022).

Enjoyable experiences also play a significant role in shaping students’ emotional experiences during PBL activities (Phung et al., 2021). Students derived enjoyment and satisfaction from engaging in project activities, especially during the phases of project discussion and execution. They had enjoyable experiences while interviewing fellow students about college life and visiting scenic spots to gather material for their projects. These enjoyable experiences provided students with satisfaction and further supported warm and collaborative relationships among peers. Students have a strong need for peer connections, which encompasses a sense of group belonging and the emotional support offered by their peers (Palmgren et al., 2021). By communicating and collaborating with their peers during the project experiences, students addressed their need for relatedness. In this study, the combination of interesting topics and enjoyable experiences significantly influenced students’ emotional engagement in the PBL process.

Behavioral engagement was enhanced more in the PBL framework than in the CTM in this study. Behavioral engagement is indicated by concentration, effort, and persistence. An explanation contributing to the enhancement of engagement in PBL classrooms could be collaborative learning. According to Chang et al. (2024), PBL promotes behavioral engagement by offering students opportunities to participate in collaborative learning. Compared to CTM, students in PBL could concentrate on learning by collaboratively planning projects, researching information and material via interviews or visiting a place, and eventually creating videos. Students were actively encouraged to engage in collaborative tasks, resulting in subsequent positive modifications in their behaviors. As reflected in the interview data, participants in PBL emphasized their active participation and concentrated on collaborative learning. Moreover, the collaborative PBL setting provided a sense of teamwork as students worked together toward a common goal. Additionally, collaborative learning played a role in helping students overcome speaking anxiety, leading to increased behavioral engagement in PBL activities. All of these elements within the collaborative activities construct an increase in behavioral engagement in PBL.

Another explanation contributes to the dynamic relationship between emotional and behavioral engagement. In the framework of student engagement proposed by Reeve (2012), there are four interrelated aspects of engagement that contribute to the experience of learning flow. Furthermore, emotional engagement is believed to be the ability to impact behavioral engagement (Reschly and Christenson, 2012). The PBL group experienced positive emotions, such as interest, enjoyment, and satisfaction in their learning from collaborative learning activities. The students were more likely to be intrinsically motivated to engage in learning activities. With the improvement of emotional engagement, they were also more likely to be attentive and focused on learning. It is well established that students achieved higher academic performance when they were emotionally involved in the educational process (Dehbozorgi and Kunuku, 2023). Consequently, as students become increasingly emotionally invested in the process of PBL, their level of behavioral engagement tends to rise correspondingly.

As for cognitive engagement, the findings show that PBL substantially enhances students’ cognitive engagement compared to CTM. Cognitive engagement is associated with the extent to which students actively employ strategic learning techniques, focusing on the use of advanced strategies rather than superficial ones (Reeve, 2013). One possible reason for the enhancement of cognitive engagement is the inquiry-based nature of PBL. PBL offers a robust framework for engaging in profound inquiry-based learning, which frequently develops students’ strategic cognitive learning. Students within the PBL context were initially motivated by an authentic problem and engaged in continuous research by asking questions and searching for answers. Eventually, they found the answers and solved the problem by employing profound learning strategies. PBL provides more opportunities for students to engage in inquiry-based learning and strategic learning (Saad and Zainudin, 2022). In contrast, the PBL setting diverges from the traditional classroom setting, where instruction is focused on the teacher and tactics are more regulated. These constrained circumstances hinder students’ ability to think or learn innovatively. Therefore, PBL students were presented with a question to explore, generate potential solutions, and develop their cognitive engagement.

Another possible reason for enhanced cognitive engagement is the activity type within the PBL framework. Zheng et al. (2022) reported that activity type, when considered as a contextual component, might have a beneficial effect on cognitive engagement in PBL. The interconnected activities in the current study, both within and outside of the classroom, allowed for greater autonomy in student-led collaboration, decision-making, problem-solving, and utilization of diverse learning strategies. Students’ cognitive engagement was enhanced by completing the series of activities. These findings align with the research conducted by Zhong et al. (2024), which demonstrated that the characteristics of various activities impact students’ cognitive engagement in PBL.

However, agentic engagement did not exhibit a similar degree of enhancement in this study compared to CTM. A key contributing factor may be the students’ limited readiness for autonomous, proactive participation in the learning process. As this was their first experience with PBL, many students were likely unfamiliar with the expectations of taking initiative, expressing preferences, or negotiating learning tasks, which are behaviors central to agentic engagement (Reeve and Tseng, 2011). Without prior scaffolding or explicit training in agentic behaviors, students may have struggled to adapt to the more student-driven structure of PBL. Furthermore, cultural norms that favor teacher authority and passive learning roles may have further hindered students’ willingness to actively shape their learning experience. These findings align with Clark and Shin (2024) study that novice PBL learners often require time and guided practice before they can comfortably assume proactive learning roles.

Additionally, the teacher’s role cannot be overlooked. Although the instructor possessed over a decade of English teaching experience and underwent PBL-related training prior to the intervention, this was her first time implementing PBL in a speaking class. According to Hidayat et al. (2024), even trained teachers often misunderstand key elements of the PBL framework, such as the role of final projects or how to scaffold student autonomy effectively. In this study, the teacher may have struggled to provide the necessary scaffolding to support students in expressing agency, particularly during early phases of the PBL cycle. This limitation may have inadvertently reinforced traditional patterns of passive learning, thus diluting the potential of PBL to enhance agentic engagement.

## 6 Conclusion, implications, limitation, and further study

This study aimed to investigate the effects of PBL in EFL students’ engagement from multiple perspectives of emotion, behavior, cognition, and agent. The findings validated that PBL could effectively enhance student engagement in speaking activities when compared to CTM. The findings further confirmed that PBL could significantly improve emotional, behavioral, and cognitive engagement compared to CTM, highlighting its value in cultivating active and meaningful student participation in English-speaking activities. Students reported increased enjoyment, stronger focus and effort, and greater use of cognitive strategies, reflecting a positive shift in their engagement patterns. However, the study found no statistically significant difference in agentic engagement between the PBL and CTM groups. These findings indicate that while PBL effectively promotes most dimensions of engagement, enhancing student agency may require additional instructional support or time for adaptation. This study provides a thorough investigation of the effectiveness of PBL in fostering behavioral, emotional, cognitive, and agentic engagement in the field of EFL in China.

This study offers implications for educators seeking to enhance students’ engagement in PBL activities. Instructors should provide consistent scaffolding to support learners’ transition to more autonomous roles, especially in contexts where PBL is novel. Incorporating real-world tasks and fostering collaborative learning environments can enhance motivation and deepen learning. Furthermore, the adaptable nature of PBL allows instructors to differentiate instruction based on individual learners’ readiness and needs, offering increased support for those unfamiliar with autonomous learning while allowing more advanced students to progress at their own pace.

Multiple limitations were identified upon completion of this research. The study was limited by its reliance on self-reported questionnaires and interviews, which may have introduced response bias to fully capture students’ engagement behaviors. Future research should adopt more varied and rigorous data collection methods, such as classroom observations, video recordings, and learner journals, to capture a richer picture of student engagement in PBL contexts. Furthermore, the novelty of PBL in the instructional setting may have affected the study outcomes, particularly with respect to agentic engagement. Both students and instructors were relatively inexperienced with the PBL model, which may have hindered the full realization of its benefits. Future studies should adopt longitudinal research designs with extended intervention periods to investigate how student engagement develops over time as both learners and instructors gain greater familiarity with the PBL approach. Cultural factors emphasizing teacher authority and passive learning may also have influenced student responsiveness to the learner-driven aspects of PBL. Future research should make cross-cultural comparisons to reveal how different educational environments influence engagement outcomes. Additionally, researchers could compare PBL with other active learning approaches (e.g., task-based language teaching, flipped classrooms, or problem-based learning) to better understand the relative effectiveness of these models in promoting holistic student engagement.

## Data Availability

The raw data supporting the conclusions of this article will be made available by the authors, without undue reservation.

## Appendix

**Table A1:** Questions asked during the semi-structured interview.

No	Questions on students’ perceived effectiveness of PBL on engagement
1	Which project themes are you mostly interested in? Why?
2	What project-based activities are you mostly interested in? Why?
3	How did you participate in the project-based activities?
4	What learning strategies did you use in the project-based activities?
5	How did you contribute to the project-based speaking class?
6	Can the project-based speaking activities help you enhance your engagement in learning? If yes, how? If no, why not?
